# An Unusual Metallic Foreign Body inside the Knee Medial Femoral Condyle

**DOI:** 10.1155/2014/849020

**Published:** 2014-11-23

**Authors:** Camilo Partezani Helito, Carlos Eduardo Nunes Faria, Marcelo Batista Bonadio, Jose Ricardo Pecora, Gilberto Luis Camanho, Marco Kawamura Demange

**Affiliations:** Department of Orthopaedics and Traumatology, Institute of Orthopedics and Traumatology-Hospital and Clinics, Faculty of Medicine, University of São Paulo (IOT-HCFMUSP), Rua Dr. Ovídio Pires de Campos, 333, Cerqueira Cesar, 05403-010 São Paulo, SP, Brazil

## Abstract

Foreign bodies in the knee joint are uncommon, particularly those not related to surgical procedures. In this paper, we present a case of an intraosseous metallic foreign body situated in the medial femoral condyle for one year, causing pain, which was removed with complete resolution of the symptoms.

## 1. Introduction

Some mechanisms that can lead to the penetration of foreign bodies into the organism are traffic accidents, explosions, or occupational accidents involving cuts in the skin [1, 2]. In these situations, the majority of the foreign bodies reported are located in the soft tissues, particularly in the subcutaneous tissue, facilitating diagnosis and access to these structures [3, 4].

One region that is more prone to the presence of foreign bodies following lesions is the foot, particularly the sole of the foot, which is subjected to constant trauma when walking [5].

Reports of intraosseous foreign bodies are more common after surgical treatment with orthopedic implants, particularly anchors, and their diagnosis outside these situations is rare [6, 7].

In this case report, we present a case of an intraosseous foreign body in the medial femoral condyle, which was only discovered one year after a trauma to the region. The foreign body was removed, with improvement of the symptoms and knee function.

## 2. Case Report

A male patient, 63 years of age, arrived at our unit with a history of pain in the medial region of the left knee for 14 months. The patient reported that the symptoms began after a trauma to the left knee with a metal disk, while he was at work. On that occasion, the disk flew off a mechanical lathe, causing a cut to the medial region of the patient's knee, at the level of the joint. The patient attended his local emergency department, where the wound was cleaned and sutured. Around twelve days after the lesion, the patient presented pain, increased volume, and inflammation in the knee and was diagnosed as having septic arthritis. He underwent open surgical drainage via the lateral parapatellar route for treatment and received antibiotics for 6 weeks, with improvement of the symptoms.

After resolution of the infection, the patient still had pain in the medial compartment of the affected knee, next to the cut caused by the initial lesion.

**Figure 1:**
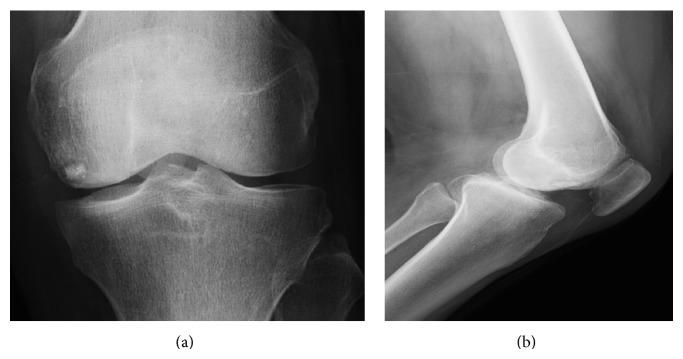
X-rays in AP (a) and profile (b) showing intraosseous metallic body in the medial femoral condyle of the left knee.

The patient entered our unit and, on physical examination, presented pain in the medial femoral condyle, without restriction of the range of movement of the knee, without ligament instabilities, with healed wounds, and without signs of infection. He underwent laboratory tests to investigate the infection, which showed normal results and X-ray of the knee, which showed an intraosseous foreign body in the medial femoral condyle, without continuity with the knee joint and without signs of affecting the adjacent cortical bone (Figure 1).

Due to the pain in this region, without any other clinical justifications, except for the foreign body, the decision was made to remove it through surgery.

**Figure 2:**
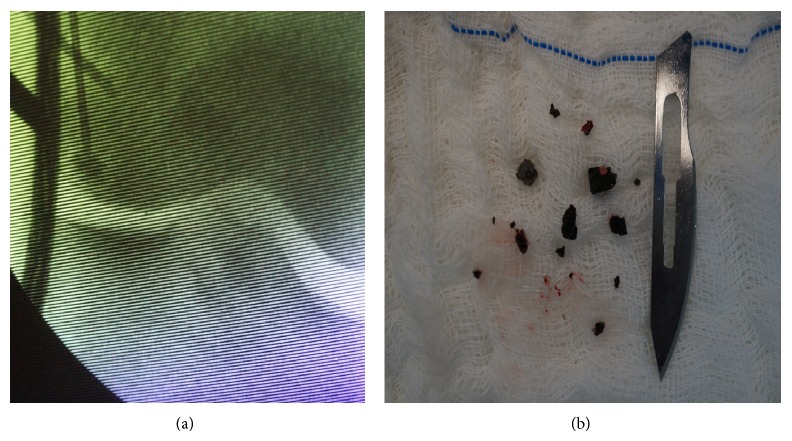
Intraoperative fluoroscopy image showing access to the foreign body via medial surgical (a) and image of the fragment removed after surgery (b).

Initially, arthroscopy was performed for intra-articular inspection of the knee. The cartilage of the femoral condyle showed normal appearance, as did the meniscal and ligament structures. Thus, it was decided to open a small bone window in the knee, via medial access, with curettage of the material, taking care not to damage the subchondral bone and joint cartilage. Fluoroscopic control was carried out to minimize damage to the subchondral bone and prevent anterograde perforation of the knee cartilage. Autologous graft of the iliac was used to fill in the missing bone after removal of the foreign bodies. The use of fluoroscopy also confirmed the complete removal of the material (Figure 2).

**Figure 3:**
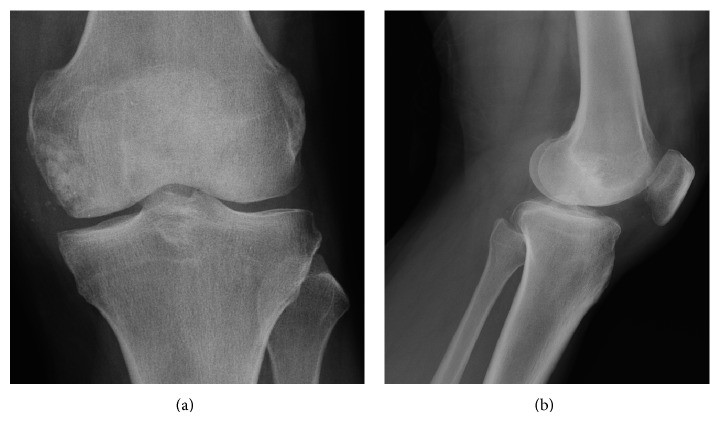
X-rays in AP (a) and profile (b) showing iliac bone graft placed in the site of the removal of the foreign body in the medial femoral condyle.

Two years after surgery, the patient presented complete healing of the incisions, consolidation of the bone graft (Figure 3), complete resolution of the pain, and normal knee function.

## 3. Discussion

Foreign bodies in the knee joint are uncommon. There are reports of foreign bodies caused by penetration of the skin or after arthroscopy. When being intra-articular, they can cause lesion of the cartilage, meniscus, or ligaments [8, 9].

In the case reported, no major intra-articular lesion occurred because the metallic body was probably intraosseous the whole time. The high impact of the initial trauma must have caused a rupture of the cortical bone of the medial femoral condyle, with the object initially becoming lodged within it. Arthroscopic inspection confirmed the absence of lesion to the cartilage at the site, which would have resulted in greater symptoms for the patient and possibly earlier discovery of the cause of the pain. The cause of the patient's pain was probably due to an inflammatory reaction of the metaphysiary bone adjacent to the piece of metal, and decompression and removal of the foreign body led to an improvement in the pain. This process is similar to that caused by osteonecrosis of the knee or of the other joints [10–12].

We believe that deep skin lesions, particularly those caused by high speed trauma or followed by joint problems, should be submitted to X-ray during the investigation. In the present case, this diagnosis might have been made in the initial evaluation if X-rays had been performed. Often, small fragments of metal or glass can lodge in the soft tissues or bones and may not be visible, even in an initial exploration of the lesion; therefore, imaging exams are important complementary tools for diagnosis.

The route used for drainage of the pyoarthritis also failed to contribute to finding the foreign body. In the joint debridement surgery, it was decided to perform a lateral parapatellar access, with probable restricted access to the medial region of the knee. An arthroscopic or medial parapatellar access route may have enabled earlier diagnosis of the foreign body [13, 14].

The case presented here is important, as we found no similar report in the literature to which we have access, with the exception of postsurgical cases, where the metal or absorbable implants used can cause granulomatous reactions or intraosseous cysts [7, 8]. In the case of metal implants in situations of postoperative follow-up, patients generally receive regular radiographic control, which facilitates the diagnosis. Even in regions more subject to the entry of foreign bodies, such as the calcaneus, only two reports were found in our search, confirming the peculiarity of the case in question [5, 15].

Due to the absence of major joint lesions and because the technique carried out preserved the joint cartilage on removal of the foreign body, the patient presented total recovery of the symptoms, with a range of movement of 0–130°, no pain, and good knee function.
